# Synthesis of coumarin or ferrocene labeled nucleosides via Staudinger ligation

**DOI:** 10.1186/1860-5397-2-23

**Published:** 2006-11-30

**Authors:** Ivana Kosiova, Andrea Janicova, Pavol Kois

**Affiliations:** 1 Comenius University, Faculty of Natural Sciences, Department of Organic Chemistry, Mlynska dolina, Pavilon CH2, SK-84215 Bratislava, Slovak Republic

## Abstract

**Background:**

Reaction of azides with triaryl phosphines under mild conditions gives iminophosphoranes which can react with almost any kind of electrophilic reagent, e.g. aldehydes/ketones to form imines or esters to form amides. This so-called Staudinger ligation has been employed in a wide range of applications as a general tool for bioconjugation including specific labeling of nucleic acids.

**Results:**

A new approach for the preparation of labeled nucleosides via intermolecular Staudinger ligation is described. Reaction of azidonucleosides with triphenylphosphine lead to iminophosphorane intermediates, which react subsequently with derivatives of coumarin or ferrocene to form coumarin or ferrocene labeled nucleosides. Fluorescent properties of coumarin labeled nucleosides are determined.

**Conclusion:**

New coumarin and ferrocene labeled nucleosides were prepared *via* intermolecular Staudinger ligation. This reaction joins the fluorescent coumarin and biospecific nucleoside to the new molecule with promising fluorescent and electrochemical properties. The isolated yields of products depend on the structure of azidonucleoside and carboxylic acids. A detailed study of the kinetics of the Staudinger ligation with nucleoside substrates is in progress.

## Background

Modified nucleosides are important tools for the study of key processes of cell metabolism, as well as being successful therapeutic agents. [1–3] For structural and functional studies of nucleosides, and their oligomers in nucleic acids or protein complexes, efficient detection methods are necessary. Today, fluorescent detection is of paramount importance to biological studies. [4–10] The sensitivity of fluorescence techniques has reached an extremely high level, similar to radioactive methods, and can even provide information on the dynamic structure of dye-bound biomolecules. Complicated and expensive optical detection would be gradually replaced by simpler and cheaper electrochemical detection based on the redox properties and electrical conductance of biomolecules. [11–12] Electrochemical techniques can be highly sensitive, rapid and available to production in miniaturised formats. Although different approaches for modification of nucleic acid components and DNA biosensor construction have been developed, many questions remain to be answered with respect to the complete understanding of optical and electrical properties of modified nucleic acids used in bioanalytical systems.

Universal starting compounds for preparation of modified nucleosides are azidonucleosides. In general, organic azides are valuable, energy-rich and flexible intermediates, which can react very differently under various reaction conditions. [13] They can react at N1 with electrophiles (carbon electrophiles, protons, boranes) and at N3 with nucleophiles, very frequently with phosphorous nucleophiles. Reaction of azides with triaryl phosphines under mild conditions gives iminophosphoranes without formation of any byproducts. [14] The intermediate which is formed almost quantitatively can be rapidly hydrolysed to the primary amine and triarylphosphine oxide. This Staudinger reduction is a frequently used method for the smooth reduction of azides to amines. Iminophosphoranes can react with almost any kind of electrophilic reagent, [15–16] e.g. aldehydes or ketones to form imines. Also less reactive carbonyl electrophiles, such as esters, can undergo reaction with iminophosphorane to form amides, especially if the electrophilic attack proceeds in an intramolecular fashion. [17–21] This so-called Staudinger ligation has been employed in a wide range of applications as a general tool for bioconjugation, [22–23] including specific labeling of nucleic acids, [24] proteomic studies [25–26] and modification of cell surfaces. [17–18]

We applied the Staudinger ligation for nucleoside labeling procedures, using coumarin and ferrocene derivatives as labels. According to our knowledge, applications of this reaction in nucleoside and nucleotide chemistry are rare.

## Results and discussion

**Figure 1:**
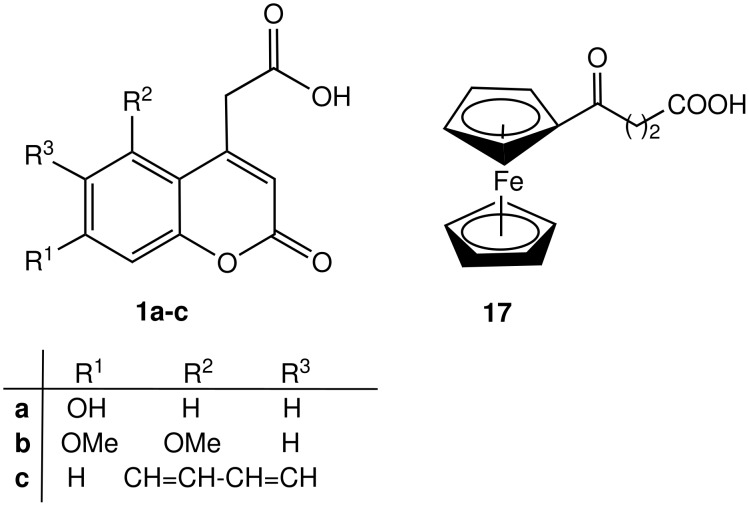
Compounds used for nucleoside labeling.

We used intermolecular Staudinger ligation [27] for the preparation of fluorescent and electrochemically labeled nucleosides. As the label we used either coumarin-4-acetic acids **1a-c** or ferrocene derivative **17** (Figure 1), which were synthesised in our department as part of studies into new ligands. Coumarins as fluorescent probes or labels [28–30] have extensive and diverse applications, they exhibit extended spectral range, are photostable and have high emission quantum yields. Ferrocene derivatives are often used as electrochemically active labels due to the accessibility of a large variety of derivatives, and their stability and easy redox tuning. [31–32]

Our key substrates were azidonucleosides, which have been used mostly as intermediates to aminonucleosides, but they also exhibit cytotoxic and antiviral properties [33] and are useful photoaffinity probes. [34] Azidonucleotides are available by several methods. [33] For preparation of 2'-azido-2'-deoxyuridine **3** we used a very convenient method starting from 2,2'-*O*-anhydrouridine. [35] 5'-Azido-5'-deoxythymidine **4** and 5'-azido-5'-deoxyuridine **5** were synthesised from 5'-tosylated intermediates, [36] whilst 3'-azido-3'-deoxythymidine **6** was prepared *via* a Mitsunobu type reaction. [37]

**Scheme 1:**
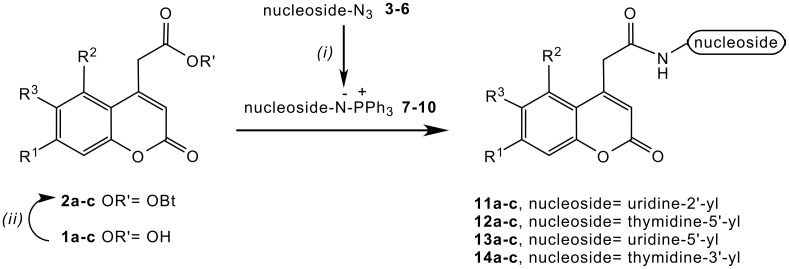
Preparation of coumarin labeled nucleosides, *(i)* PPh_3_, acetonitrile, *(ii)* HOBT, DCC, dioxane

**Figure 2:**
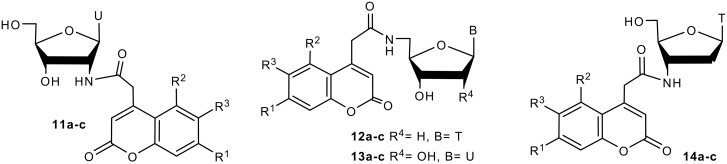
Coumarin labeled nucleosides prepared by intermolecular Staudinger ligation.

Staudinger reaction of azidonucleosides **3–6** with triphenylphosphine led to iminophosphorane intermediates **7–10**, which reacted subsequently with active esters of coumarin-4-acetic acids **2a-c** (Scheme 1) to form amide bond of new nucleoside derivatives **11–14** (Figure 2). [see Supporting Information File 1] It is known that the Staudinger ligation is accelerated in polar solvents, [21] thus we made our experiments in a mixture of acetonitrile and dioxane. The overall reaction rate was high enough at temperatures around 0°C in all cases (1–2 hours reaction time), but at temperatures around -20°C reactions were too slow. The conversion of coumarin-4-acetic acid active esters was almost quantitative in all cases, according to TLC.

**Scheme 2:**
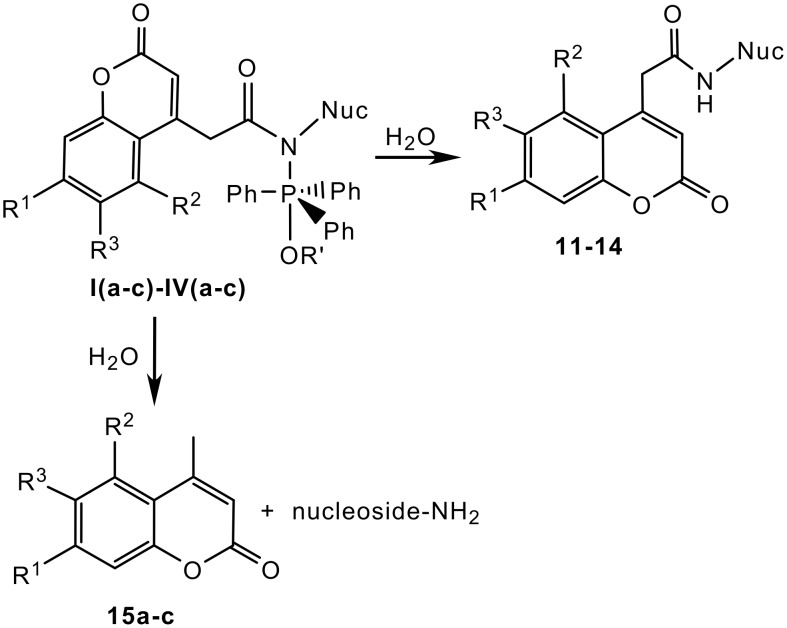
Hydrolysis of proposed intermediates **I(a-c)-IV(a-c)**

The yield of desired products in Staudinger ligations depends on the structure of the carboxylic acid and on the azido group position on the nucleoside. Addition of water to the proposed intermediates **I-IV** (Scheme 2) resulted not only in the formation of the desired products, but also in the formation of 4-methyl coumarins **15a-c** and aminonucleosides depending on the substrates used. The aminonucleosides and derivatives **15a-c**, as products of a concurrent reaction, complicated the monitoring and the work-up of the reaction mixture. The R_f_ values of the aminonucleosides and iminophosphorane derivatives **7–10** are very similar and therefore the progress of reaction was monitored and the conversion of substrates was calculated based on coumarin derivatives **2a-c**.

The relationship between the yield of desired product and the azidonucleoside structure is not straightforward. Starting from 2'-azido-2'-deoxyuridine **3** or 5'-azido-5'-deoxythymidine **4** we prepared desired modified nucleosides **11a-c** and **12a-c** in good yields (Table 1). 4-Methyl derivatives of the corresponding coumarin-4-acetic acids were only minor byproducts in these reactions. The isolated yields of polar products were negatively influenced by lengthy separation by flash chromatography. The structures of the products were confirmed by ^1^H and ^13^C NMR, ^1^H-^1^H COSY and ^13^C-^1^H HSQC analysis. The signal due to the NH-function of the newly created amide bond was clearly visible in all cases. We expected analogous results in the reactions of 5'-azido-5'-deoxyuridine. Surprisingly, the reactions of this 5'-azidonucleoside with coumarin derivatives **2a-c** gave products in low yields, max. 15%. We isolated derivatives **15a-c** as the main products in these cases. According to RP HPLC analyses the reaction mixture contained also 5'-amino-5'-deoxyuridine along with small amount of starting compounds **1a-c**. The structure of products **13a-c** was confirmed by ^1^H NMR analysis. The ratio of product and 4-methyl derivative is probably influenced by diverse effects of nucleobase on the reaction mechanism, especially the interaction of nucleobases with triphenylphosphine residue in the intermediates **I(a-c)-IV(a-c)**. Similar behaviour was observed in the case of 3'-azido-3'-deoxythymidine **6**. Reaction led to the exclusive formation of 4-methyl derivatives **15a-c** and 3'-amino-3'-deoxythymidine, and changes to the standard reaction conditions did not induce the formation of the desired 3'-labeled nucleosides **14a-c**.

**Table 1:** Isolated yields and spectral characteristics of coumarin labeled nucleosides **11**–**12** ^a^

*Compound* ^b^	*Yield (%)*	λ*_ex_(nm)*	ε *(cm*^-1^*M*^-1^*)*	λ*_em_(nm)*	*FI (A.U.)*

**1a**	-	326	12487	392	1955
**11a**	64	326	9981	395	2202
**12a**	48	325	7123	392	1923
**1b**	-	323	3032	418	2260
**11b** ^c^	59	324	9033	428	2420
**12b**	41	322	10006	429	2373
**1c**	-	318/349	7444	417	719/930
**11c**	56	319/350	4713	418	822/1006
**12c**	44	319/350	5291	416	876/1224

^a^ products **13a-c** were isolated in low yields (max. 15%) and products **14a-c** were not isolated ^b^ c = 10^-4^ M in methanol ^c^ c = 0.5 × 10^-4^ M in methanol

The spectral characteristics of the isolated products are summarised in Table 1. We found that all newly prepared conjugates display only one peak in the fluorescence spectrum in methanol. The process of conjugation did not cause a shift in the absorption and fluorescence maxima of our compounds, in comparison with maxima of **1a-c.** We observed only slight changes of fluorescence intensity in the series of coumarin labeled nucleosides.

**Scheme 3:**
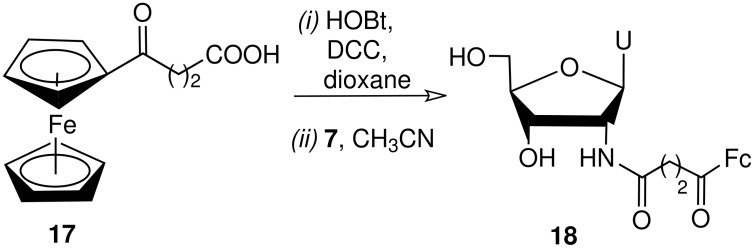
Preparation of ferrocene labeled uridine

We also tested the Staudinger ligation as a prospective method for the electrochemical labeling of nucleosides and nucleotides with ferrocene derivatives. 2'-Azido-2'-deoxyuridine **3** was chosen as an azidosubstrate for its satisfactory reactivity in previous experiments and the 2' position of carbohydrate residues is a versatile site for chemical modification of nucleosides. The ferrocene derivative (4-ferrocenyl-4-oxobutanoic acid) **17** was used as an electrochemical label. Analogous reaction of 4-ferrocenyl-4-oxobutanoic acid reactive ester with iminophoshorane nucleoside derivative **7** gave 2'-ferrocene labeled uridine (Scheme 3). The yield of pure isolated product **18** was 57% and its structure was confirmed by ^1^H and ^13^C NMR, ^1^H-^1^H COSY and ^13^C-^1^H HSQC analysis.

## Conclusion

We successfully applied the Staudinger ligation to the synthesis of labeled nucleosides. For this purpose, we prepared several types of azidonucleoside substrates and carboxylic acid derivatives. Although the conversion of reactants was almost quantitative in all cases, the yield of Staudinger ligation was found to be dependent on the structure of the azidonucleoside and the carboxylic acids. A detailed study of the kinetics of the Staudinger ligation with nucleoside substrates, and the possible application to the construction of labeled oligomers and novel bioconjugates for enzyme assays is in progress. A very useful way to use Staudinger ligation in parallel syntheses could be the application of solid-phase reagents to simplify lengthy purification of products.

## Supporting Information

File 1 Experimental Section
