# Supplementary material for: Synthesis of coumarin or ferrocene labeled nucleosides via Staudinger ligation
Source: Beilstein J Org Chem. 2006 Nov 30;2:23. doi: 10.1186/1860-5397-2-23 (PMC1779791; doi:10.1186/1860-5397-2-23)
Supplement: File 1 — Experimental Section [file Beilstein_J_Org_Chem-02-23-s001.pdf]

Supplementary material

Synthesis of coumarin or ferrocene labeled nucleosides via Staudinger ligation

Ivana Kosiova*, Andrea Janicova, Pavol Kois

Comenius University, Faculty of Natural Sciences, Department of Organic Chemistry, Mlynska dolina, Pavilon CH2, SK-84215 Bratislava, Slovak Republic

kosiova@fns.uniba.sk, phone: +0421 (0) 2 60296 338

janicova@fns.uniba.sk, phone: +0421 (0) 2 60296 338

kois@fns.uniba.sk, phone: +0421 (0) 2 60296 341

*corresponding author

Experimental section

General methods

Solvents were distilled and dried *via* established methods.[1] Melting points were measured on a Kofler hot stage and are uncorrected. NMR spectra were recorded in DMSO with Varian VX UNITY spectrometer (300 MHz/75 MHz for $^{1}H/^{13}C$). Chemical shifts are referenced to Me_4Si (^{1}H) or the residual solvent signal (^{13}C). All measurements were run at room temperature. The ^{1}H and ^{13}C assignments were based on ^{1}H-^{1}H COSY, ^{13}C-^{1}H HSQC and ^{13}C-^{1}H HMBC experiments. Analyses of reaction mixtures and products were performed by RP HPLC on LC 5000 Ingos, column Lichrospher® 100, RP C8 (5 µm, 25 cm, ø 0.4 cm). Absorption spectra were recorded using UV-VIS spectrophotometer Agilent 8453, cuvette lenght 1 cm. Fluorescence spectra were recorded using Hitachi F-2000. Infrared spectra were obtained on Perkin-Elmer Spectrum GX FT-IR spectrometer with ATR sampling accessory. TLC were performed on precoated plates of silica gel 60 F_{254} (Merck) using the eluent A (chloroform/methanol 9/1, v/v) or eluent B (acetone/ethanol/ethyl acetate/water 1/1/6/0.5, v/v) as eluent. The crude products were purified using column chromatography on silica gel using the chloroform/methanol as eluent. 3'-Azido-3'-deoxythymidine[2] **3** 5'-azido-5'-deoxyuridine[3] **4**, 5'-azido-5'-deoxythymidine[3] **5** and 2'-azido-2'-deoxyuridine[4] **6** were prepared as described in the literature. CAUTION: The handling of low-molecular weight azides is dangerous, due to their potentially explosive character. Neat azidonucleosides must not be heated and all reactions should be carried out on a small scale. 2-(2-oxo-2*H*-chromen-4-yl)acetic acids were prepared as described in literature.[5]

General procedure

The final reaction mixture was prepared by addition of mixture I to the cooled mixture II. **Mixture I:** Azidonucleoside (1 molar. equiv.) was dissolved in dry CH_3CN and PPh_3 (1.1 molar. equiv.) was added. Reaction mixture was stirred at r.t until substrate consumed, as judged by TLC (eluent A) and was used imediatelly in third step.

Mixture II: Coumarin-4-acetic acid (1.0 molar equiv.) was dissolved in dry dioxane, HOBt (1.1 molar equiv.) and DCC (2.0 molar equiv.) was added. Reaction mixture was stirred at r.t. until substrate consumed, as judged by TLC (eluent A), to gave reactive ester of carboxylic acid. Then mixture II was dilluted with dry CH_3CN and mixture was cooled at -15˚C. After dropwise addition of mixture I to the cooled mixture II, the final I+II mixture was stirred until the temperature reached r.t., and until the reactive coumarin acid ester has consumed, as judged by TLC (eluent A).

The mixture was evaporated to dryness after quenching with water and the products were separated by column chromatography in methanol/chloroform (5/95-40/60, v/v).

2-(7-Hydroxy-2-oxo-2*H*-chromen-4-yl)-*N*-(uridin-2'-yl)acetamide (11a)

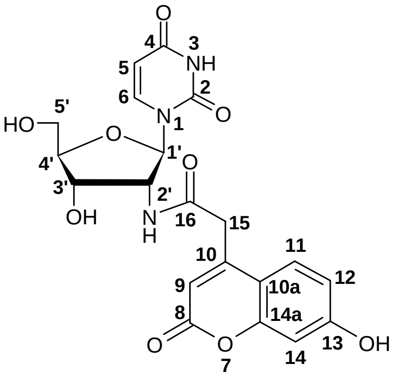

White solid product (105 mg, 0.24 mmol, 64%), m.p.= 185-189˚C, R_f= 0.49 (B), $C_{20}H_{19}N_3O_9$ (445.38), calculated: 53.93% C, 4.30% H, 9.43% N, 32.33% O, found: 54.05% C, 4.28% H, 9.25% N, **^{1}H NMR (DMSO):** 11.27 (1H, s, H-3), 8.37 (1H, d, $J_{2',NH}$= 8.8 Hz, NH-CO), 7.88 (1H, d, $J_{5,6}$= 8.3 Hz, H-6), 7.48 (1H, d, $J_{11,12}$= 8.8 Hz, H-11), 6.77 (1H, dd, $J_{12,14}$= 2.5 Hz, $J_{11,12}$= 8.8 Hz, H-12), 6.70 (1H, d, $J_{12,14}$= 2.5 Hz, H-14), 6.14 (1H, s, H-9), 5.99 (1H, d, $J_{1',2'}$= 8.5 Hz, H-1'), 5.64 (1H, d, $J_{5,6}$= 8.2 Hz, H-5), 4.52 (1H, m, H-2'), 4.09 (1H, m, H-3'), 3.96 (1H, m, H-4'), 3.74 (2H, s, H-15), 3.58 (2H, m, H-5'); **^{13}C NMR (DMSO):** 168.43 (16), 162.96 (4), 161.10 (8), 160.17 (13), 154.86 (10), 151.24 (2), 150.84 (14a), 140.62 (6), 126.58 (11), 113.05 (9), 111.91 (12), 111.72 (14), 102.18 (5), 86.80 (1'), 85.49 (4'), 70.56 (3'), 61.58 (2'), 54.49 (5'), 30.69 (15); **IR (neat):** 3260, 1688, 1680, 1608, 1568, 1394, 1270, 1140, 1059, 824.

2-(5,7-Dimethoxy-2-oxo-2*H*-chromen-4-yl)-*N*-(uridin-2’-yl)acetamide (11b)

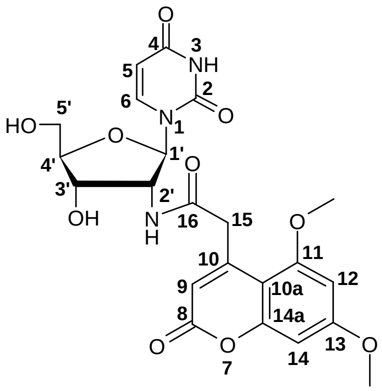

White solid product (106 mg, 0.22 mmol, 59%), m.p. = 190˚C-deccomposed, R_f= 0.63 (B), $C_{22}H_{23}N_3O_{10}$ (489.43), calculated: 53.99% C, 4.74% H, 8.59% N, 32.69% O, found: 53.82% C, 4.91% H, 8.38% N, **^{1}H NMR (DMSO):** 11.29 (1H, s, H-3), 8.03 (1H, d, $J_{NH,2'}$= 8.6 Hz, NH-CO), 7.88 (1H, d, $J_{5,6}$= 8.3 Hz, H-6), 6.58 (1H, d, $J_{12,14}$= 2.0 Hz, H-12), 6.43 (1H, d, $J_{12,14}$= 2.2 Hz, H-14), 6.01 (1H, s, H-9), 5.97 (1H, d, $J_{5,6}$= 8.6 Hz, H-5), 5.64 (1H, d, $J_{1',2'}$= 7.9 Hz, H-1’), 4.54 (1H, m, H-2’), 4.10 (1H, m, H-3’), 3.94 (1H, m, H-4’), 3.83 (3H, s, CH_3O-11), 3.78 (2H, s, H-15), 3.72 (3H, s, CH_3O-13), 3.57 (2H, m, H-5’), **^{13}C NMR (DMSO):** 169.38 (16), 162.99 (8), 162.65 (4), 159.71 (13), 158.31 (11), 156.22 (10), 151.15 (14a), 150.94 (2), 140.87 (6), 113.06 (9), 103.70 (10a), 102.11 (5), 95.35 (14), 93.57 (12), 86.58 (1’), 85.46 (4’), 70.57 (3’), 61.59 (2’), 55.91 (CH_3O-11), 55.60 (CH_3O-13), 54.06 (5’), 42.59 (15); **IR (neat):** 3281, 1690, 1608, 1567, 1553, 1463, 1394, 1273, 1141, 1091, 1046, 811.

2-(3-Oxo-3H-benzo[f]chromen-1-yl)-*N*-(uridin-2’-yl)acetamide (11c)

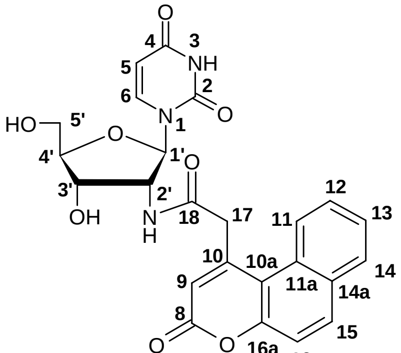

Light yellow solid product (101 mg, 0.27 mmol, 56%,), m.p.= 186-190˚C, R_f= 0.69 (B), $C_{24}H_{21}N_3O_8$ (479.44), calculated: 60.12% C, 4.41% H, 8.76% N, 26.70% O, found: 60.21% C, 4.22% H, 8.52% N, **^{1}H NMR (DMSO):** 11.38 (1H, s, H-3), 8.55 (1H, d, $J_{5,6}$= 8.7 Hz, H-6), 8.31 (1H, d, $J_{NH,2'}$= 8.4 Hz, NH-CO), 8.18 (1H, d, $J_{15,16}$= 9.1 Hz, H-15), 8.03 (1H, dd, $J_{11,12}$= 7.8 Hz,

$J_{12,13}$= 1.7 Hz, H-12), 7.80 (1H, d, $J_{13,14}$= 7.9 Hz, H-14), 7.62 (1H, dd, $J_{13,14}$= 7.5 Hz, $J_{12,13}$= 1.7 Hz, H-13), 7.57 (1H, d, $J_{11,12}$= 7.4 Hz, H-11), 7.55 (1H, d, $J_{15,16}$= 8.90 Hz, H-16), 6.52 (1H, s, H-9), 6.05 (1H, d, $J_{5,6}$= 8.7 Hz, H-5), 5.88 (1H, m, HO-4'), 5.54 (1H, d, $J_{1',2'}$= 7.9 Hz, H-1'), 5.17 (1H, m, HO-5'), 4.56 (1H, m, H-2'), 4.41-4.15 (2H, m, H-5'), 4.08 (1H, m, H-3'), 3.96 (1H, m, H-4'), 3.56 (2H, s, H-17), **^{13}C NMR (DMSO):** 168.73 (18), 162.90 (4), 159.20 (10), 154.07 (8), 151.86 (2), 151.04 (16a), 140.59 (6), 133.83 (11a), 130.79 (15), 129.41 (14a), 129.06 (14), 127.91 (12), 125.57 (13), 124.56 (11), 117.86 (16), 117.39 (10a), 113.85 (9), 102.18 (5), 86.67 (1'), 85.11 (4'), 70.65 (3'), 61.58 (2'), 50.01 (5'), 43.30 (17); **IR (neat):** 3290, 1729, 1671, 1546, 1264, 1090, 1065, 1030, 863, 805, 754.

2-(7-Hydroxy-2-oxo-2*H*-chromen-4-yl)-*N*-(thymidin-5'-yl)acetamide (12a)

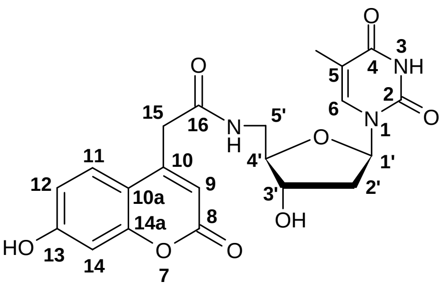

Light yellow solid product (96 mg, 0.22 mmol, 48%), m.p.= 250-255˚C, R_f = 0.54 (B), $C_{21}H_{21}N_3O_8$ (443.4), calculated: 56.88% C, 4.77% H, 9.48 % N, 28.87 % O, found: 56.59% C, 4.83% H, 9.52 % N, **^{1}H NMR (DMSO):** 11.29 (1H, s, H-3), 8.46 (1H, t, $J_{5',NH}$= 6.6 Hz, NH-CO), 7.57 (1H, d, $J_{11,12}$= 8.2 Hz, H-11), 7.42 (1H, s, H-6), 6.74 (1H, dd, $J_{11,12}$= 8.6 Hz, $J_{12,14}$= 2.3 Hz, H-12), 6.70 (1H, d, $J_{12,14}$= 2.3 Hz, H-14), 6.16 (1H, s, H-9), 6.16 (1H, t, $J_{1',2'}$= 7.2 Hz, H-1'), 5.33 (1H, m, HO-3'), 4.13 (1H, m, H-3'), 3.77 (1H, m, H-4'), 3.68 (2H, s, H-15), 3.30 (2H, m, H-5'), 2.08 (2H, m, H-2'), 1.69 (3H, s, H_3C-5); **^{13}C NMR (DMSO):** 168.08 (16), 163.66 (4), 161.22 (8), 160.24 (13), 154.96 (10), 151.23 (14a), 150.45 (2), 136.01 (6), 126.72 (11), 112.76 (12), 111.71 (9), 111.40 (11a), 109.73 (5), 102.24, 84.75 (4'), 83.86 (1'), 71.23 (3'), 41.25 (15), 38.42 (5'), 33.35 (2'), 11.88 (CH_3-5); **IR (neat):** 3282, 1689, 1676, 1609, 1567, 1460, 1394, 1269, 1138, 1059, 857, 809.

2-(5,7-Dimethoxy-2-oxo-2*H*-chromene-4-yl)-*N*-(thymidin-5'-yl)acetamide (12b)

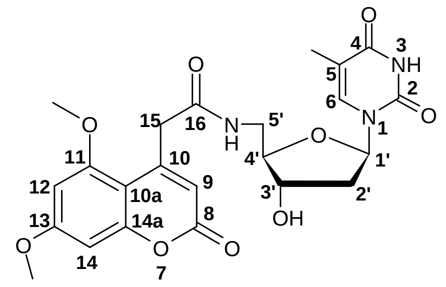

Light yellow solid product (78 mg, 0.16 mmol, 41%), m.p.= 150-154°C, R_f = 0.65 (B), $C_{23}H_{25}N_3O_9$ (487.46), calculated: 56.67% C, 5.17% H, 8.62% N, 29.54% O, found: 56.78% C, 5.14 % H, 8.38% N, **^{1}H NMR (DMSO):** 11.28 (1H, s, H-3), 8.09 (1H, t, $J_{NH,5'}$= 6.1 Hz, HN-CO), 7.46 (1H, s, H-6), 6.57 (1H, d, $J_{12,14}$= 2.2 Hz, H-14), 6.41 (1H, d, $J_{12,14}$= 2.0 Hz, H-12), 6.16 (1H, t, $J_{1',2'}$= 6.1 Hz, H-1'), 6.06 (1H, s, H-9), 4.13 (1H, m, H-3'), 3.83 (3H, s, H_3C-13), 3.80 (1H, m, H-4'), 3.76 (3H, s, H_3C-11), 3.74 (2H, s, H-15), 3.35 (2H, m, H-5'), 2.07 (2H, m, H-2'), 1.68 (3H, s, H_3C-5); **^{13}C NMR (DMSO):** 168.95 (16), 163.69 (4), 162.65 (8), 159.80 (13), 158.38 (11), 156.28 (10), 151.12 (14a), 150.45 (2), 136.07 (6), 113.20 (9), 109.72 (5), 103.71 (10a), 95.20 (14), 93.54 (12), 84.83 (4'), 83.89 (1'), 71.38 (3'), 55.91 (CH_3O-11), 55.88 (CH_3O-13), 42.81 (15), 41.36 (5'), 38.35 (2'), 11.84 (CH_3-5); **IR (neat):** 3302, 1689, 1674, 1609, 1565, 1394, 1270, 1213, 1138, 1061, 819, 756.

2-(3-Oxo-3*H*-benzo[f]chromen-1-yl)-*N*-(thymidin-5'-yl)acetamide (12c)

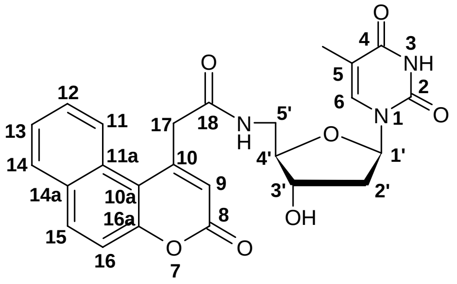

Light yellow solid product (81 mg, 0.17 mmol, 44%), m.p. = 217-221°C, R_f = 0.67 (B), $C_{25}H_{23}N_3O_7$ (477.47), calculated: 62.89% C, 4.86% H, 8.80% N, 23.46% O, found: 62.67% C, 4.32% H, 8.76% N, **^{1}H NMR (DMSO):** 11.23 (1H, s, H-3), 8.56 (1H, t, NH-CO), 8.18 (1H, d, $J_{15,16}$= 8.8 Hz, H-15), 8.02 (1H, dd, $J_{11,12}$= 7.7 Hz, $J_{12,13}$= 1.1 Hz, H-12), 7.84 (1H, dd, $J_{13,14}$= 8.3 Hz, $J_{12,13}$= 1.1 Hz, H-13), 7.60 (1H, d, $J_{13,14}$= 8.5 Hz, H-14), 7.57 (1H, d, $J_{15,16}$= 9.1 Hz, H-16), 7.52 (1H, d, $J_{11,12}$= 6.7 Hz, H-11), 7.37 (1H, d, $J_{6,Me}$= 0.8 Hz, H-6), 6.58 (1H, s, H-9), 6.19 (1H, t, H-1'), 5.32 (1H, d, HO-3'), 4.23 (2H, s, H-18), 4.13 (1H, m, H-3'), 3.78 (1H, m, H-4'), 3.37 (2H,

m, H-5'), 2.04 (2H, m, H-2'), 1.53 (3H, s, CH_3-5); **^{13}C NMR (DMSO):** 168.34 (18), 163.58 (4), 159.24 (8), 154.14 (10), 151.82 (2), 150.46 (16a), 135.99 (6), 130.82 (11a), 129.99 (15), 129.51 (14), 129.12 (14a), 127.91 (12), 125.31 (13), 124.83 (11), 118.02 (16), 117.41 (9), 113.91 (10a), 109.63 (5), 84.65 (1'), 83.75 (4'), 71.28 (3'), 43.56 (17), 41.38 (5'), 38.36 (2'), 11.71 (CH_3-5); **IR (neat):** 3283, 1676, 1608, 1567, 1460, 1394, 1269, 1080, 1060, 999, 818.

4-(Ferrocen-1-yl)-4-oxo-*N*-(uridin-2'-yl)butanamide (18)

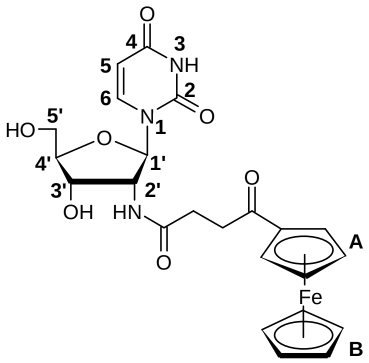

Orange oil (88 mg, 0.16 mmol, 57%), R_f = 0.45 (A), $C_{25}H_{30}FeN_3O_7$ (540.4), calculated: 55.57% C, 5.60% H, 10.33% Fe, 7.78% N, 20.73 % O, found: 55.42% C, 5.78% H, 7.54% N ; **^{1}H NMR (DMSO):** 11.29 (1H, s, H-3), 7.89 (1H, d, $J_{5\text{-}6}$= 8.2 Hz, H-6), 7.87 (1H, d, $J_{NH,2'}$= 8.8 Hz, NH-CO), 5.94 (1H, d, $J_{1'\text{-}2'}$= 8.8 Hz, H-1'), 5.65 (1H, d, $J_{5\text{-}6}$= 8.0 Hz, H-5), 5.18 (1H, m, H-2'), 4.77 (2H, m, Fc), 4.53 (2H, m, Fc), 4.25 (5H, s, Fc), 4.1 (1H, m, H-3'), 3.94 (1H, m, H-4'), 3.58 (1H, m, H-5'), 2.94 (2H, t, CH_2-but), 2.52 (2H, t, CH_2-but), **^{13}C NMR (DMSO):** 201.99 (C=O, but), 172.26 (NH-C=O, but), 163.14 (4), 150.95 (2), 140.88 (6), 102.02 (5), 86.71 (1'), 85.68 (4'), 78.70 (Cp A), 71.87 (Cp A), 70.61 (3'), 69.06 (Cp B), 69.01 (Cp A), 61.68 (2'), 54.43 (5'), 34.21 (but), 28.87 (but); **IR (CCl_4):** 3736, 2961, 2341, 1807, 1698, 1672, 1461, 1411, 1310, 1097, 1051, 1030, 814, 663.

References

1. Armarego, W.L.F.; Chai, C. *Purification of Laboratory Chemicals,* Butterworth-Heinemann, an imprint of Elsevier Science, 5th Edition, 2003
2. Kirschenheuter, G. P.; Zhai, Y.; Pieken, W. A. *Tetrahedron Lett.* 1994, **35:**8517-8520.
3. Ciuffreda, P.; Loseto, A.; Santaniello, E. *Tetrahedron* 2002, **58:**5767-5771.
4. Czernecki, S.; Valery, J.-M. *Synthesis* 1991, 239-240.
5. Charitos, C.; Kokotos, G.; Tzougraki, C. *J. Heterocycl. Chem.* 2001, **38:**153-158.
